# Adrenocortical carcinoma presenting with heterosexual pseudoprecocious puberty shortly after birth: case report and review

**DOI:** 10.3332/ecancer.2013.289

**Published:** 2013-01-29

**Authors:** F Ghazizadeh, M Ebadi, S Alavi, MT Arzanian, B Shamsian, F Jadali

**Affiliations:** 1 Pediatric Congenital Hematologic Disorders Research Center, Shahid Beheshti Medical University, Tehran, Iran; 2 Tehran University of Medical Sciences, Tehran, Iran; 3 Department of Pathology, Mofid Children’s Hospital, Shahid Beheshti Medical University, Tehran, Iran

**Keywords:** adrenocortical carcinoma, pseudoprecocious puberty, virilisation, paediatric

## Abstract

Adrenocortical tumour is rare in children. We report on a female infant with adrenocortical carcinoma presenting with pseudoprecocious puberty at the age of two. She had a history of gradually increasing public hair growth after birth. Physical examination showed signs of virilisation such as pubic hair growth and hirsutism with evidence of facial hair growth. On biochemical evaluation, DHEA-S, 17-OH progesterone, and testosterone levels were elevated. An abdominopelvic spiral computed tomography (CT) scan with intravenous contrast identified a well-defined heterogeneously enhanced mass with areas of necrosis in the right adrenal gland and downward displacement of the underlying kidney. There was no evidence of distant metastasis on CT imaging. An exploratory laparotomy was performed in which a large, haemorrhagic and necrotic mass in the right adrenal gland with pressure effect on right liver lobe and signs of thrombosis in the inferior vena cava was detected. Pathologic examination confirmed the adrenocortical carcinoma. She received eight cycles of adjuvant chemotherapy with Carboplatin, Etoposide, and Doxorubicin regimens and underwent follow-up visits thereafter in which no sign of recurrence was observed. In conclusion, adrenocortical carcinomas are rare in children, but they should be considered in any child presenting with signs of pseudoprecocious puberty.

## Introduction

Adrenocortical tumours (ACTs) are rare in children, with a worldwide incidence of 0.3–4 cases per million each year in children younger than 15 years of age. However, incidence shows a broad geographic variation, ranging from 0.3–0.38 cases per million each year in Europe and the United States to 3.4–4.3 cases per million each year in Brazil [1]. The first case of a paediatric ACT was reported in 1865 [1]. These tumours show a preponderance in the female population, and bilaterality has been reported in 2%–10% of cases [2]. They derive from the adrenal cortical cells and may produce steroids, autonomously. Unlike adult ACTs, paediatric ACTs are functional and hormonally active in about 90% of the cases [3], presenting with virilisation, pseudoprecocious puberty or Cushing syndrome [4].

ACTs include both adrenocortical adenomas (ACAs) and adrenocortical carcinomas (ACCs) [2], with carcinomas being less common, comprising approximately 0.2% of all paediatric cancers; only 25 new cases/year in the United States are being reported [2, 5]. The distinction between adenoma and carcinoma is of great importance and depends on the presence or absence of certain pathologic criteria and/or clinical parameters [6]. Herein, we report on a two-year-old female with ACC presenting with pseudoprecocious puberty. To the best of our knowledge, this is the first reported case from Iran.

## Case report

**Figure 1:**
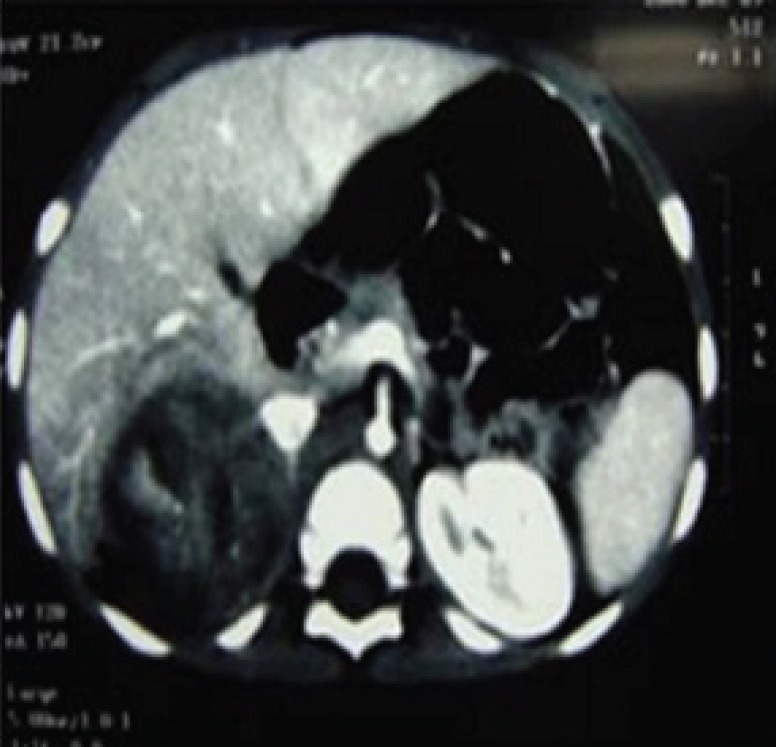
An abdominopelvic CT scan image shows a large oval shaped heterogeneous mass arising from the right adrenal gland with heterogeneous enhancement and pressure effect on right lobe of the liver.

A two-year-old girl presented with pubic hair growth from birth that showed gradual increase. Linear growth was normal; she was normotensive without cushingoid features. At initial examination, signs of the virilisation and hirsutism with increased facial hair growth were prominent. There were no neurocutaneous marks. Examination of her genitalia revealed thick pubic hair, mild clitoromegaly, and hypertrophy of the labia major. She had a history of admission for neonatal hypoglycemia. Abdominopelvic ultrasound (US) revealed a hypoechoic mass measuring 55 × 39 mm in the right suprarenal area. She was then referred to the oncology clinic. An abdominopelvic spiral computed tomography (CT) scan with intravenous contrast identified a well-defined heterogeneously enhanced mass with areas of necrosis in the right adrenal site with downward displacement of the underlying kidney (Figure 1). A filling defect in the inferior vena cava (IVC) was evident and suggested tumour thrombus with extension into the IVC. No focal lesion was observed in the liver. A chest CT scan was normal. Initial laboratory investigations revealed normal haematological and biochemical parameters. According to the signs and symptoms and imaging findings, a hormonally active ACT was suspected; thus hormonal studies were performed and the results were as follows: dihyrdroepiandrosterone sulphate (DHEA-S) 900 μg/dL (normal range: 5–85 μg/dL), 17-OH progesterone 4.2 ng/mL (normal: <2.0), and testosterone 2.4 ng/mL (normal range: 0.01–0.2 ng/mL). Although there were no clinically evident signs of Cushing syndrome, plasma levels of cortisol and urinary free cortisol levels were measured and found to be within the normal range. There was no evidence of bone and bone marrow metastasis as investigated by a whole body bone scan and bone marrow aspiration with biopsy, respectively.

**Figure 2:**
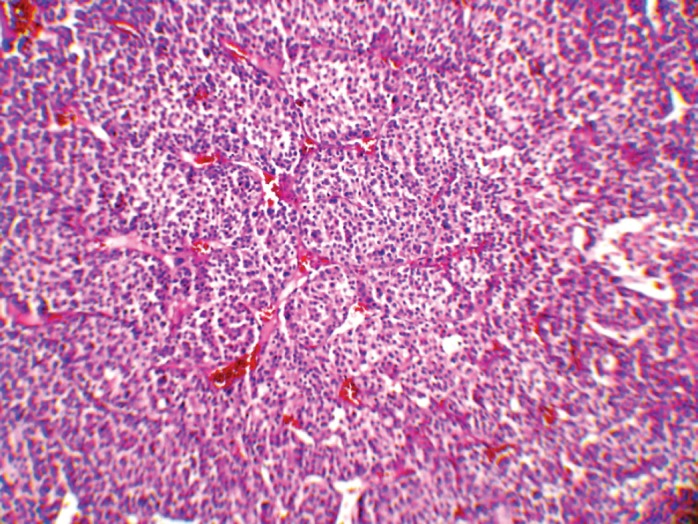
A low-magnification (10x) pathology image showing the neoplasm composed of atypical cells with pleomorphic nuclei and abundant oeosinophilic cytoplasm arranged in sheets with vascularised stroma.

The patient underwent an exploratory laparotomy through which a large haemorrhagic and necrotic mass replacing the whole adrenal gland on the right side was detected. Signs of local invasion to the right liver lobe and thrombosis in the IVC were observed during the surgery, with no evidence of regional lymphadenopathy. An adrenalectomy was performed for the patient. On gross appearance, there were multiple irregular fragile tissue fragments, with areas of cystic change, in total measuring 12 × 7 × 3 cm (252 cm^3^) and weighing around 90 g. A microscopic examination revealed large atypical cells with pleomorphic atypic nuclei, prominent nucleoli, and moderate oeosinophilic cytoplasm arranged in a lobular and solid pattern (Figure 2). Extensive necrosis, high mitotic activity with atypical mitoses, and both vascular and capsular invasions were evident. Based on the Children’s Oncology Group (COG) classification (Table 1) and extra adrenal invasion, the tumour was considered as Stage III. The patient had no signs of distant metastasis. She was scheduled to receive a combination chemotherapy with Carboplatin, Etoposide, and Doxorubicin for eight cycles every three weeks. During the treatment, the patient was assessed by clinical examination every three weeks (with each chemotherapy cycle) biochemical evaluations and abdominopelvic CT/magnetic resonance imaging (MRI) were performed at the three-month follow-up visits. The patient’s hormone profile normalised in three months, whereas the virilisation demonstrated gradual improvement and resolved in six months. The patient was followed for three years and remained in a complete remission with no sign of recurrence.

## Discussion

Our patient was diagnosed at the age of two, which is in the range of peak incidence years of ACT occurrence. The predominant feature of this case that is worth mentioning is the appearance of pubic hair since birth. In contrast to paediatric carcinomas in general, which are associated with a progressive increase in incidence with age, ACTs have a peak incidence between years one and four, followed by a decline to 0.1 per million during the subsequent ten years, then it rises to 0.2 per million and finally reaches another peak during the fifth decade of life [7]. According to the Surveillance, Epidemiology and End Results Program, 19-20 new cases of adrenocortical carcinoma in children and adolescents per year occur in the United States [8]. ACTs in children are rare and, interestingly, have less aggressive clinical behaviour when compared with their adult counterparts [3, 9]; the tumour in our patient did not behave aggressively, and the case had a good outcome. This pattern of incidence and the tumour’s tendency to be predominantly androgen producing in this age group could be linked to the tumour’s origin in the predominantly androgen-producing foetal adrenal cortex [8], as suggested by the histopathologic features of ACTs in young children [7]. These tumours are predominant in female children, and the female–male ratio is 2:1 [6]. Our patient was a two-year-old Caucasian girl. Worldwide, the incidence of ACTs varies across geographic regions; in children younger than 14 years of age, the reported incidence per million ranges from 0.1 in Hong Kong and Bombay to 0.4 in Los Angeles and to 3.4 in Southern Brazil, indicating a ten times higher incidence in Brazil [1–3]. However, there is no report regarding the incidence of ACTs in the Middle East. Nonetheless, paediatric ACTs appear to have a consistent clinical course despite their diversity in geographic, ethnic, environmental, and possibly genetic characteristics [7]. Our patient presented with features of heterosexual *pseudoprecocious puberty, *mainly the virilisation, which is known as the most common manifestation of adrenocortical carcinoma in children; although, in our case, linear growth was normal, biochemical evidence of increased levels of dihyrdroepiandrosterone sulphate, 17-OH progesterone, and testosterone were compatible with a functionally active virilising tumour; we think that the increased amount of virilising hormones to this level resulted in an earlier diagnosis, before the tumour reached higher weight and larger size. In this case, 17-OH progesterone remained elevated even after the tumour resection, normalising after three months postoperatively. These tumours can be associated with overproduction of other adrenal hormones, including glucocorticoids [7]. In our case, classic features of glucocorticoid excess, including growth failure, hypertension, acne, plethora, buffalo hump, and excessive weight gain were all absent, and biochemical evidence of excessive glucocorticoid production was not observed. The right suprarenal hypoechoic mass was confirmed by ultrasonography with CT scan. CT scan and ultrasonography are the imaging techniques most routinely used for diagnosis of ACTs, with CT scan being more sensitive in terms of localisation and identification [10].

**Figure 3:**
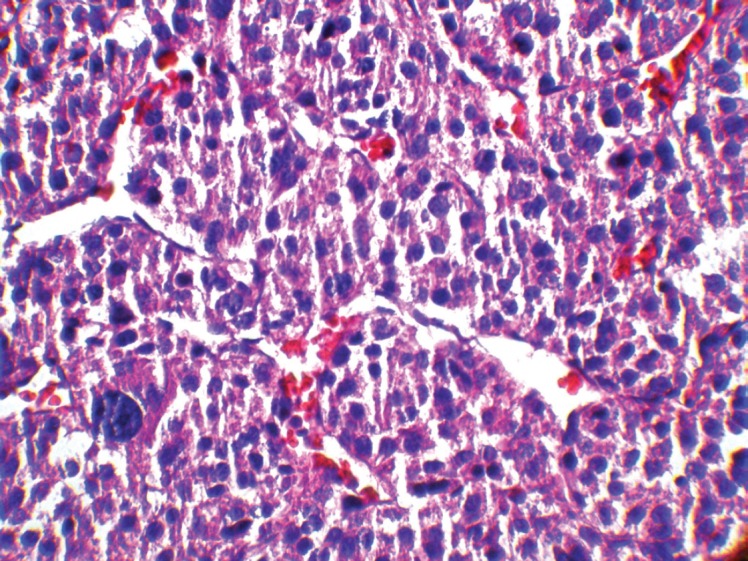
A high-magnification (40x) pathology image: the tumour cells are highly pleomorphic with hyperchromatic large nuclei and oeosinophilic cytoplasm.

It has been shown that in paediatric ACTs, the disease stage, age at presentation, signs of endocrine dysfunction, and tumour weight and volume are important prognostic markers [6]. The COG staging system is used for staging ACTs in children [10]; our case, due to persistent postoperative elevation of 17-OH progesterone and extra adrenal invasion, was considered as Stage III. In our patient, invasion of the liver and IVC and high mitotic activity with atypical mitoses were observed, each indicating malignancy independently. A large, haemorrhagic, and necrotic mass was observed during exploratory laparotomy. Areas of necrosis and haemorrhage are frequent in carcinomas and very rare in adenomas. Invasion of major veins is a frequent finding in carcinoma and often leads to total occlusion, thrombosis and embolism. All of these findings existed in our patient. On gross appearance, multiple fragile fragments weighing 90 g in total, were observed; however, a tumour weight of >100 g is usually considered as malignant [12] and the observed tumour size is recognised as a prognostic factor [10] [13]. Other features indicating a malignant adrenal tumour (adrenal carcinoma) in our patient (according to Weineke’s definition of malignancy) included large atypical cells, pleomorphism, atypical nuclei, prominent nucleoli, moderate oeosinophilic cytoplasm arranged in a lobular and solid pattern, and finally capsular and vascular invasion (Figure 3). It has been shown that Weineke’s criteria lack sensitivity and specificity, and their predictive value is not confirmed yet [9]. A study reported that the common presentation was hormone-related symptoms [3]; this is in concordance with our case as our patient was reported with the virilisation, pubic hair, and hirsutism with increase in facial hair growth. Interestingly, capsular invasion and necrosis, which are both well-defined unfavourable criteria in the grading system of Weiss for adult ACTs, were concurrently present in our patient. As described by West *et al*, six- to eight-fold lower levels of human leukocyte antigen (HLA)-DRB1, HLA-DPB1, HLA-DRA and HLA-DPA1 are detected in paediatric adrenal carcinomas compared with adenomas on gene expression profiling analysis [3]. Mutation in the p^53 ^gene is estimated to occur in 80%–90% of all paediatric ACTs. Mutation of TP53 R337H, a distinct germline of p^53^, s described in Brazilian patients with ACT [5]. Unlike their adult counterparts, the expression of MMP2 or the loss of HLA-class II antigens in paediatric ACTs does not discriminate between malignant and benign tumours. Because of the high costs of genetic analysis, we stayed within the limits of detecting the mutation. Different treatment modalities are considered on the basis of the stage of the tumour. Treatment basically consists of surgical resection and mitotane- or cisplatin-based chemotherapy [7] per the standard treatment modality for this stage of the disease. Our patient underwent a total adrenalectomy on the right followed by chemotherapy including Cisplatin, Etoposide and Doxorubicin for a period of eight courses and received monthly follow-up visits thereafter for three years. The prognosis in our patient was favourable and no sign of relapse was evident during follow-up.

## Conclusion

The ACT should be considered in any child with virilising features and pseudoprecocious puberty, as it is the most predominant presentation. To achieve a favourable outcome in paediatric ACTs, early diagnosis, careful preoperative examinations including biochemical tests, abdominopelvic, and chest CT scans with bone scans. and finally performing complete adrenalectomy followed by adjuvant chemotherapy and postoperative follow-up visits are recommended.

## Figures and Tables

**Table 1.** COG Staging of Adrenocortical Tumors in Children

Stage	Symptoms
I	Completely resectable, small tumors (<100 g and <200 cm^3^) with normal postoperative hormone levels
II	Completely resectable, large tumors (≥100 g or ≥200 cm^3^) with normal postoperative hormone levels
III	Unresectable, gross or microscopic residual disease Tumor spillage Patients with stages I and II of tumors fail to normalise hormone levels after surgery Patients with retroperitoneal lymph node involvement
IV	Presence of metastatic disease
